# Heterogeneous base catalysts for edible palm and non-edible Jatropha-based biodiesel production

**DOI:** 10.1186/1752-153X-8-30

**Published:** 2014-05-03

**Authors:** Hwei Voon Lee, Joon Ching Juan, Nurul Fitriyah Binti Abdullah, Rabiah Nizah MF, Yun Hin Taufiq-Yap

**Affiliations:** 1 Nanotechnology & Catalysis Research Centre (NanoCat), Institute of Postgraduate Studies, University Malaya, 50603 Kuala Lumpur, Malaysia; 2 Catalysis Science and Technology Research Centre, Faculty of Science, Universiti Putra Malaysia, 43400 UPM Serdang, Selangor, Malaysia

**Keywords:** Transesterification, Palm oil, Jatropha oil, Solid base catalyst, Alkaline-earth metal oxide, Mixed metal oxides

## Abstract

**Background:**

Transesterification catalyzed by solid base catalyst is a brilliant technology for the noble process featuring the fast reaction under mild reacting condition in biodiesel production. Heterogeneous base catalysts are generally more reactive than solid acid catalysts which require extreme operating condition for high conversion and biodiesel yield. In the present study, synthesis of biodiesel was studied by using edible (palm) or non-edible (Jatropha) feedstock catalyzed by heterogeneous base catalysts such as supported alkali metal (NaOH/Al_2_O_3_), alkaline-earth metal oxide (MgO, CaO and SrO) and mixed metal oxides catalysts (CaMgO and CaZnO).

**Results:**

The chemical characteristic, textural properties, basicity profile and leaching test of synthesized catalysts were studied by using X-ray diffraction, BET measurement, TPD-CO_2_ and ICP-AES analysis, respectively. Transesterification activity of solid base catalysts showed that > 90% of palm biodiesel and > 80% of Jatropha biodiesel yield under 3 wt.% of catalyst, 3 h reaction time, methanol to oil ratio of 15:1 under 65°C. This indicated that other than physicochemical characteristic of catalysts; different types of natural oil greatly influence the catalytic reaction due to the presence of free fatty acids (FFAs).

**Conclusions:**

Among the solid base catalysts, calcium based mixed metal oxides catalysts with binary metal system (CaMgO and CaZnO) showed capability to maintain the transesterification activity for 3 continuous runs at ~ 80% yield. These catalysts render high durability characteristic in transesterification with low active metal leaching for several cycles.

## Background

Industrialization processes continue to grow globally in par with human population which leads to the growing worldwide demand for energy as well as for petrochemical resources, coal and natural gases. This phenomenon has caused the depletion rate of fossil energy resources to increase exponentially and caused alarming environmental problems to the society. Recently, many fuel developers have showed interests in alternative renewable fuels to substitute or blend with petroleum-based fuels. An alternative fuel shall be easily available, environment friendly and techno-economically competitive [1,2].

Biodiesel plays a major role in energy sector due to its similar combustion properties with petroleum. Furthermore, biodiesel is sometimes more superior than petroleum diesel with improved physical and chemical properties, such as higher flash point, higher cetane number, ultralow sulfur content, better lubricity, improved biodegradability, and smaller carbon footprint [3-5]. Chemically, biodiesel is a mixture of methyl esters with long-chain fatty acids and is typically made from transesterification reaction of biological triglyceride sources such as vegetable oil and animal fats with alcohol in the presence of catalyst. This process reduces the viscosity to a value comparable to that of diesel and hence improves combustion [6].

According to Meng et al. (2009), biological feedstock supply for biodiesel production covers more than 75% of the overall production cost [7]. The favorable properties in selecting the best biodiesel feedstock include lowest oil price, high oil content, favorable fatty acid composition (saturated or unsaturated acid), low cultivation maintenance and costs, controllable growth and harvesting season, consistent seeds maturity rates and potential market for agricultural by-products [8].

In general, biodiesel feedstock can be divided into 4 main categories which are: (a) edible vegetable oil, (b) non-edible vegetable oil, (c) waste or recycled oil and (d) animal fats [9]. The most common feedstock employed in biodiesel production is edible and inedible oil from oleaginous plants grown in different regions. Soybean oil, sunflower oil, rapeseed oil and palm oil have been used as edible feedstock in biodiesel synthesis [10]. In Malaysia, biodiesel production is synonymous to palm oil as oil palm plantations possesses higher productivity per hectare of oil palm with lowest oil production cost per unit as compared to other vegetable oils like rapeseed and soybean [11,12]. A hectare of oil-palm plantation produces approximately 3.62 tonnes/ha/year of oil, 5–9 times higher than other oil producing crops like soybean, sunflower and rapeseed, which produces 0.4, 0.46 and 0.68 tonnes/ha/year, respectively [13]. This keeps the price of palm-based biodiesel competitive enough to meet the demand of commodity market [9,14].

Instead of edible palm oil, non-edible feedstock is getting interest as a biodiesel feedstock in biodiesel production. Amongst the varieties of non-edible plant oil, Jatropha is the most favorable for biodiesel production as they meet the major requirement of biodiesel standards of USA, Germany and European Standard Organization. Jatropha crops can be well adapted to arid and semiarid conditions like non-cropped marginal lands and waste lands with harsh environments. Hence, the cultivation cost is lower as these crops can still sustain reasonably high yield without intensive care. Besides, Jatropha oil possesses similar composition as other vegetable oil, which favors biodiesel production. In addition, Jatropha oil contains of toxic phorbol ester which is unsuitable for human consumption and thus reduces the competitive with food supply market [15,16].

A new type of heterogeneous catalysis technology has been developed to adapt the natural characteristics of biodiesel feedstock and existing transesterification technology. Utilization of heterogeneous catalyst for biodiesel production has offered some relief to biodiesel producers by improving their ability to process alternative and cheaper feedstock with simplified processes and cheaper manufacturing processes with prolonged catalyst lifetime. The three factors namely catalytic activity, catalyst life and oil flexibility have tremendous impact on the cost of biodiesel [3,17]. According to some research studies, the heterogeneous catalysts used for palm-based and Jatropha-based biodiesel productions are mainly from solid base catalysts e.g. alkali metal supported catalyst, hydrotalcite, alkaline-earth metal oxides, mixed metal oxides and natural waste shell, which render high transesterification activity with > 80% of biodiesel yield. It was realized that the main criteria to catalyze transesterification of these biodiesel feedstock with base catalysts is low FFA <3 wt.% and moisture 1 wt.% in the feedstock to avoid from unfavorable side reaction such as oil hydrolysis and saponification [18-27]. The formation of soap was observed in base catalyzed transesterification of high acid oil with low biodiesel yield.

By drawing on this, an attempt has been made in the present work to produce biodiesel from edible (palm) and non-edible (Jatropha) oils using heterogeneous base catalysts: (a) supported alkali metal catalysts– sodium hydroxide supported with alumina (NaOH/Al_2_O_3_), (b) alkaline-earth metal oxides– magnesium oxide (MgO), calcium oxide (CaO) and strontium oxide (SrO) and (c) calcium-based mixed metal oxides– (CaMgO and CaZnO). This study was aimed to investigate the versatility of solid base catalysts with different chemical characteristic for transesterification of edible and non-edible biodiesel feedstock. The physicochemical properties (chemical composition, textural properties and basicity) of synthesized solid base catalysts produced thru wet impregnation and co-precipitation techniques were investigated. Furthermore, effects of the catalyst loading and reaction time towards catalyst activity were investigated in order to optimize transesterification conditions. The reusability of the solid catalysts was evaluated by batch experiment and the reasons for the deactivation of the catalyst were also discussed by performed the catalyst leaching test.

## Results and discussion

### Catalysts characterization

**Figure 1:**
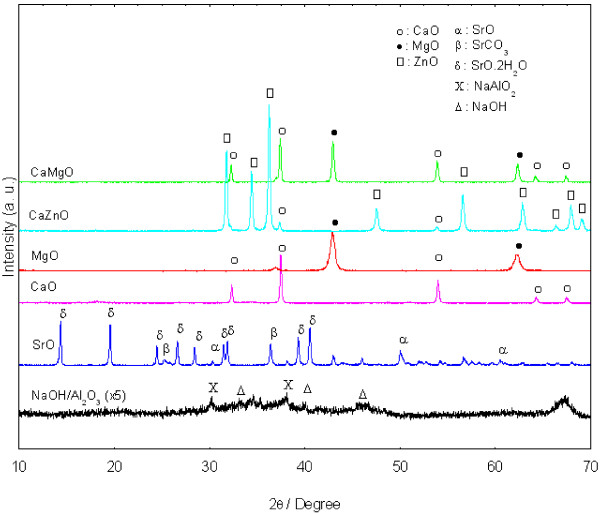
XRD diffractogram of synthesized solid base catalysts.

The crystalline structure of alumina supported alkali metal (NaOH/Al_2_O_3_), alkaline-earth metal oxide (CaO, MgO, SrO) and calcium-based mixed metal oxides (CaMgO and CaZnO) base solid catalysts was revealed by X-ray diffraction pattern (Figure 1). The diffraction peaks of NaOH/Al_2_O_3_ showed the presence of NaOH (JCPDS File No. 27-0711) and sodium aluminate (NaAlO_2_) phases (JCPDS File No. 20-1073). The intensity of Al_2_O_3_ peaks were reduced when NaOH was introduced into Al_2_O_3_ support, this is due to well dispersion of NaOH on the Al_2_O_3_ support in the form of monolayer covering the surface of support. The results indicate that NaOH has reacted with Al_2_O_3_ thus giving rise to the formation of aluminates and is in good agreement with that reported by Arzamendi’s and Kim’s research groups [28,29]. The diffractogram of MgO clearly showed that the presence of oxide phases at 2θ of 42.9 and 62.2° (JCPDS File No. 4-0829). For CaO, the diffraction pattern depicted intensified peaks at 2θ angles of 32.2, 37.3 and 53.8° (JCPDS File No. 37-1497). SrO catalyst showed main reflection peaks at 2θ = 30.31, 50.54° and 63.04° (JCPDS File No. 1-074-1227). And the other peaks observed indicated the presence of SrCO_3_ and SrO.2H_2_O phases (JCPDS File No. 1-084-1778 and 28-1222). Strontium compound is difficult to regenerate by thermal treatment, and their decomposition temperatures are higher than 1200°C [30]. Comparing to MgO and CaO, SrO can be easily absorbed by CO_2_ and hydrolyzed by moisture to form carbonate and hydroxide phase, respectively which compatible with the XRD result. The XRD patterns of calcium-based mixed metal oxides catalysts recorded the patterns of both samples (CaMgO and CaZnO) were corresponded to pure oxide, and no new crystalline phase attributable to the formation of mixed oxides could be detected. CaMgO catalyst showed the appearance of cubic CaO and hexagonal MgO phases where CaZnO catalyst also gave diffraction peaks of CaO and wurzite structure of ZnO phase at 2θ angles of 31.7°, 34.4° and 36.2° (JCPDS File No. 36-1451).

The textural properties of the solid base catalysts with various calcination temperatures were determined by BET measurement (Table 1). Results showed that BET surface area of pure γ-Al_2_O_3_ is 136 m^2^/g. However, the surface area of the NaOH/γ-Al_2_O_3_ catalyst was decreased to 52 m^2^/g after NaOH impregnation and thermal activation. This reflects a significant reduction of pore volume of Al_2_O_3_ penetrated by Na^+^ ion as shown in Table 1[28]. The surface area and porosity of alkaline-earth metal oxide: MgO, CaO and SrO catalysts vary with different thermal activation temperature of 600, 800 and 1200°C, respectively. Calcination at high temperature is required to thermally activate the metal carbonate salts to active metal oxide catalyst for transesterification reaction, especially for SrO catalyst which > 1000°C of heating is required to remove the strong bonding of carbonate from the catalyst’s surface. The surface area and pore volume for MgO, CaO and SrO were decreased as the calcination temperature increased, whereby the pore diameter were increased with temperature as the pore structure collapsed with high temperature. For calcium-based mixed oxides catalysts: CaMgO and CaZnO, the textural properties are apparently small when synthesized using coprecipitation method. The precursor of binary metal-based catalyst in carbonate form required high temperature (800°C) to render pure active mixed oxide phase for reaction which led to sintering effect of catalysts.

**Table 1:** Surface area profile of synthesized base catalysts

**Synthesized catalyst**	**Calcination temperature (°C)**	**BET area (m**^**2**^**/g)**	**Pore volume (cm**^**3**^**/g)**	**Pore diameter (Å)**
Alkali supported alumina				
Al_2_O_3_	-	136	0.436	132.6
NaOH/Al_2_O_3_	500	52	0.235	139.1
Alkaline-earth metal oxide				
MgO	600	29.7	0.132	160.5
CaO	800	9.5	0.072	213.6
SrO	1200	1.0	0.005	216.0
Calcium-based mixed metal oxides				
CaMgO	800	15.5	0.103	225.3
CaZnO	800	9.8	0.098	220.4

**Figure 2:**
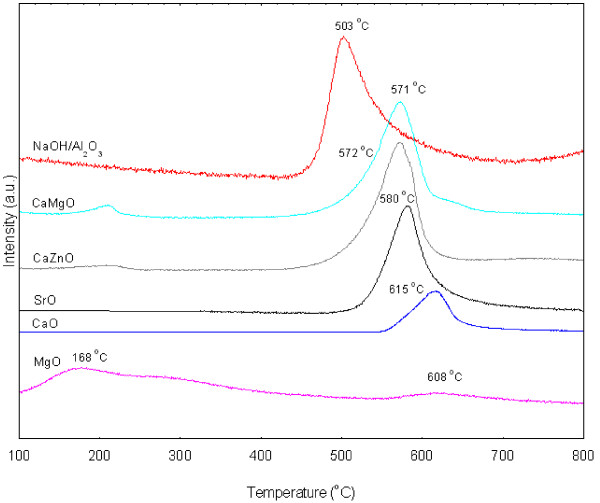
**TPD-CO**_**2 **_**spectra of synthesized solid base catalysts.**

Several studies have reported that basicity of catalyst is the major key to improve the transesterification activity. Among basicity distribution strength (weak, medium and strong basic strength), the number of strong basic sites that are considered to be the main parameter in yielding a high biodiesel yield [31]. The total basicity and basic site distributions of synthesized solid base catalysts were measured by temperature programmed desorption of CO_2_ (Table 2 and Figure 2). Figure 2 showed the different desorption peaks of catalysts, which indicated the presence of basic sites with different basic strengths. The CO_2_ desorption band at 100–500°C are assigned as interaction of CO_2_ with sites of weak and medium basic strengths. Whereas, the CO_2_ desorbed at temperature of ~ 600°C can be attributed to the much stronger basic site corresponding to unbounded O^2-^ anions [32,33]. The results showed that calcium-based mixed metal oxides (CaMgO and CaZnO) and alkali supported alumina (NaOH/Al_2_O_3_) possess similar number of basic sites (~400 μ mol of CO_2_/g). This indicated that catalyst with multi-metal ion interaction showed synergy effect by enhancing the basicity on active site of catalyst [34]. However, the basicity of single metal oxide catalysts (MgO, CaO and SrO) were lower than binary metal catalyst as absence of combination base properties between two base metal oxides. From the results, SrO showed highest amount of basicity albeit lower amount of surface area than MgO and CaO. This is in agreement with Hattori’s theory which stated that increase of basic characteristic of oxide when going down Group II elements (Mg, Ca and Sr metal cation) [35]. In addition, the surface area of Group II oxides were in reverse sequence with basicity order, this implies that surface area is not as main key to promoting the basicity and transesterification rate [36]. The TPD-CO_2_ profile of CaO showed the desorption peak at 615°C, which slightly higher compared to the desorption temperature of calcium-based mixed metal oxides catalyst means (~570°C). This could be explained that higher temperature of heating is required to desorb the strong interaction of CO_2_ from the active basic sites of CaO. The presence of strong basic strength in single CaO or binary system of calcium-based catalyst formed strong bonding with acidic CO_2_, and thus higher temperature (minimum > 500°C) require for desorption process. From Table 2, although both CaO and Calcium-based catalyst render strong basic strength, the binary metal oxide provide higher amount of strong basic active sites as compared to CaO catalyst.

**Table 2:** **TPD-CO**_**2 **_**profile of synthesized base catalysts**

	**Amount of basic site (μ mol of CO**_**2**_**/g)**			**Total basicity**
**Catalyst**	**T**_**max **_**(<300°C)**	**T**_**max **_**(300–500°C)**	**T**_**max **_**(>500°C)**	**(μ mol of CO**_**2**_**/g)**
Alkali supported alumina				
NaOH/Al_2_O_3_	-	-	467.68	467.68
Alkaline-earth metal oxide				
MgO	31.36	-	18.95	50.31
CaO	-	-	290.42	290.42
SrO	-	-	396.56	396.56
Calcium-based mixed oxide				
CaMgO	2.68	-	449.70	452.38
CaZnO	-	-	412.70	412.70

### Transesterification of edible palm and non-edible Jatropha oils

The transesterification of edible (palm) and non-edible (Jatropha) oils in the presence of different solid base catalysts (NaOH/Al_2_O_3_, MgO, CaO, SrO, CaMgO and CaZnO) were studied thoroughly by varying the transesterification conditions. The transesterification reaction was performed at different reaction time (2–6 h) and catalyst amount (1–5 wt.%) under constant reflux temperature (65°C) and methanol to oil ratio of 15:1.

**Figure 3:**
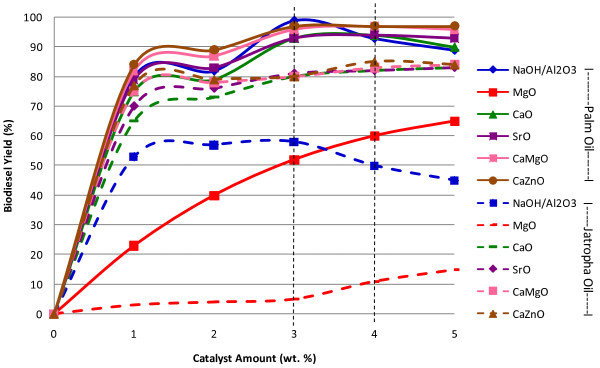
Effect of catalyst amount for solid base catalyzed transesterification reaction of palm and Jatropha oil (methanol: oil ratio of 15:1, 65°C and 3 h).

**Figure 4:**
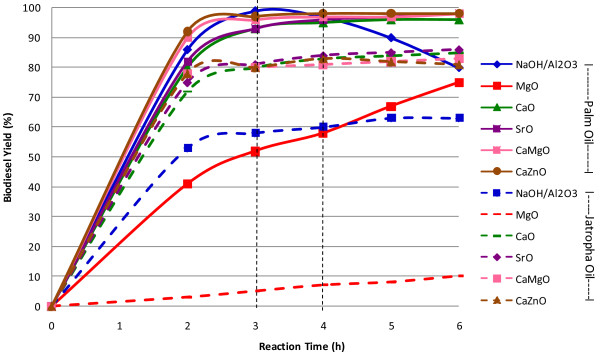
Effect of reaction time for solid base catalyzed transesterification reaction of palm and Jatropha oil (methanol: oil ratio 15:1, 65°C, 3 wt.%).

The catalyst amount is a crucial factor to improve the rate of transesterification reaction. As reported by most of the studies, the increase of catalyst loading shall increase surface active sites of catalyst. This indirectly improves transesterification process by increase the accessibility of triglyceride and methanol to the catalyst surface. Other than that, reaction time for transesterification is another important criterion to determine the equilibrium point of reaction in order to avoid any reverse process [6,37]. The biodiesel profile revealed that edible palm oil underwent transesterification relatively at a higher reaction rate compared to the non-edible Jatropha oil. Results showed that the transesterification of palm oil yielded more than 90% of biodiesel under mild condition of 3 wt.% catalyst, 3 h reaction time and 15:1 methanol/oil molar ratio. Jatropha biodiesel production required higher amount of catalyst (4 wt.%) (Figure 3) and longer reaction time (4 h) (Figure 4) in order to improve the biodiesel yield to more than 80% compared to palm biodiesel.

**Figure 5:**
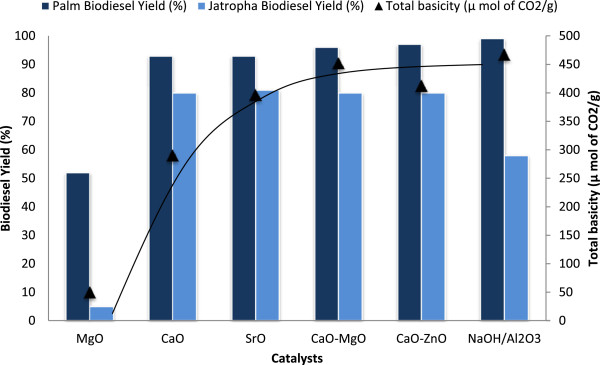
Correlative effect between catalysts basicity and transesterification activity (at 3 wt.% of catalyst, 3 h, 15:1 methanol: oil ratio and 65°C).

The present study imply that the nature of edible and non-edible oil significantly influence the transesterification activity of catalysts [38]. Although non-edible Jatropha oil has tremendous potential for biodiesel production but it contains high free fatty acids (FFAs) as compared to palm oil. The FFAs content is key parameter for determining the viability of the vegetable oil in transesterification process. Higher FFAs content of the oil will lower the biodiesel selectivity. This happened to NaOH/Al_2_O_3_ catalyzed transesterification of high acid Jatropha oil. Jatropha biodiesel yield is 58%, while the low acid palm oil achieved conversion of 99% (Figure 5). The presence of FFAs in Jatropha oil triggered saponification with sodium base catalyst resulting soap formation which could lead to formation of gel and emulsion. This is highly undesirable and complicates the purification process of biodiesel [10]. The NaOH/Al_2_O_3_ was deactivated as the active sodium was loss to formation of soap and in turn reduced transesterification activity. FFAs content directly influences biodiesel yield in biodiesel production.

Other than oil characteristics, the rate of transesterification is greatly depending on catalyst’s basicity. Among the solid base catalysts, the catalytic activity of MgO for both palm and Jatropha-based transesterification are low, which is 52% and 5%, respectively. The results suggested that, under reflux condition, the optimum content of biodiesel synthesis using edible and non-edible oil greatly depend on the type of the solid base catalyst used. According to Di Serio and his co-researcher [33], basicity of catalyst is directly proportional to the biodiesel yield. MgO catalyst with lower amount of basicity is less active than other catalysts by producing less yield of biodiesel under mild condition. This fact has been proven by the correlation effect between transesterification activities and basicity of different catalysts (Figure 5).

### Durability of catalyst

**Figure 6:**
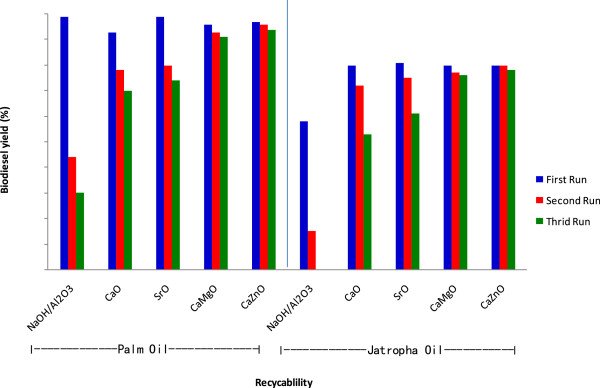
Reusability of solid base catalysts.

Under optimized conditions (reaction temperature of 65°C, 3.0 wt.% of catalyst, 15:1 methanol/oil molar ratio and 3 h reaction time), the solid base catalysts (NaOH/Al_2_O_3_, CaO, SrO, CaMgO and CaZnO) were reutilized for another two runs for both edible palm and non-edible Jatropha oil (Figure 6). Among these catalysts, CaMgO and CaZnO demonstrated high reusability. However, MgO catalyst was not selected for the reusability test as it is low in catalytic activity. Surprisingly, only calcium based mixed metal oxides catalysts are capable to maintain high yield > 80% of palm-biodiesel and > 70% of Jatropha-biodiesel at third run. From the results, NaOH/Al_2_O_3_ catalyzed reaction drop drastically throughout the three runs. This apparently demonstrated that the catalyst was unstable during reaction in which Na-O-Al was decomposed in the present of methanol in the first run reaction medium. Therefore, the catalytic activities of second and third runs were reduced as the active Na species was reduced. For alkaline-earth metal oxides catalysts, the catalytic activities were reduced gradually in every cycle. The metal oxides are poisoned by absorption of H_2_O, CO_2_ and reaction medium (e.g. glycerol, oil, methyl ester). Extra washing step and thermal activation is required prior to each cycle to maintain high reusability. In order to evaluate the leaching of the catalyst, elemental analysis was performed to study the leaching of Ca, Mg and Zn phases for CaMgO and CaZnO catalysts. The result for the fresh and used catalyst was depicted in Table 3. It is showed that the only minor amount of calcium, magnesium and zinc leached out during reaction and thus it is negligible. This result is concordance with the catalytic activity of CaMgO and CaZnO which capable of maintaining it activity after the third cycles.

**Table 3:** Leaching tests profile of CaMgO and CaZnO

**Catalysts**	**CaMgO**		**CaZnO**	
**Element content**^**a **^**(ppm)**	**Ca**	**Mg**	**Ca**	**Zn**
Fresh catalyst	17.9 ± 0.5	36.2 ± 0.3	10.0 ± 0.4	74.3 ± 0.7
3rd run ^b^	15.6 ± 0.7	29.8 ± 0.2	8.6 ± 0.3	67.4 ± 0.6

^a^Ca, Mg and Zn content from catalysts were determined by ICP-AES analysis.

^b^Leaching experiments were conducted in triplicate to determine the content of the CaMgO and CaZnO after reaction in Jatropha oil.

## Experimental

### Preparation of solid base catalysts

#### Supported alkaline metal catalysts: NaOH/Al_2_O_3_

The catalyst was prepared by incipient wetness impregnation of γ-Al_2_O_3_ powder with an aqueous solution of NaOH compounds. Al_2_O_3_ support was impregnated with 15 ml sodium hydroxide (50 wt.%) solution. The impregnate was dried in oven at 100°C overnight and undergone thermal treatment at 500°C.

#### Alkaline-earth metal oxide: MgO, CaO and SrO

MgO, CaO and SrO were obtained after calcination of pulverized magnesium carbonate (MgCO_3_), lime stone (CaCO_3_) and strontium carbonate (SrCO_3_).

#### Mixed metal oxide: CaMgO and CaZnO

The mixed metal oxide catalysts were prepared by using co-precipitation method, and subsequent calcinations of the precursors. These catalysts were obtained by slowly adding a 2 M aqueous solution of the corresponding metal nitrates to an aqueous solution containing Na_2_CO_3_ and NaOH. The precipitation was performed under vigorous stirring at 65°C, for 1 day. Finally, the solids were filtered, washed with deionized water and dried at 100°C. The synthesized precursors were then undergone thermal treatment (800°C) in the air to produce mixed metal oxides.

### Characterization

The crystalline phases of synthesized catalysts were analyzed by powder X-ray diffraction analysis using a Shimadzu diffractometer model XRD 6000 with employing Cu-K_α_ radiation to generate diffraction patterns from powder crystalline samples at ambient temperature. Specific surface area of the catalysts was obtained by the BET (Brunauer-Emmer-Teller) method using Thermo Finnigan Sorptomatic 1900 series nitrogen adsorption/desorption analyzer. The total basicity and basic strength of the catalysts were measured by temperature programmed desorption of carbon dioxide (CO_2_-TPD) using a Thermo Finnigan TPDRO 1100 apparatus provided with a thermal conductivity detector. The elemental composition of fresh and used catalysts was determined by using inductively coupled plasma-atomic emission spectrometer (ICP-AES) analysis that conducted by using Perkin Elmer Emission Spectrometer Model Plasma 1000.

### Catalytic test

The biodiesel feedstock, palm oil and Jatropha oil were purchased from local market and Bionas Sdn. Bhd., respectively. Physicochemical properties of both palm and Jatropha oil were shown in Table 4. Catalytic activity was evaluated by performing transesterification reaction in a batch reflux-reactor, which palm or Jatropha oil was mixed with methanol and synthesized solid base catalyst. The mixture was added into round-bottom flask equipped with a reflux condenser and heated at 65°C under constant stirring speed at 800 rpm. Investigation on effect of reaction time (2–6 h) and catalyst amount (1–5 wt.%) was investigated in order to achieve optimum biodiesel yield. Upon the completion of reaction, the product was cooled to ambient temperature and the catalyst was separated thru centrifugation. The excessive methanol was removed using rotary evaporator. Reaction product was then poured into a separation funnel and left overnight for separation with upper layer as biodiesel and glycerin at the bottom layer. The biodiesel product was analyzed by using a gas chromatography (PerkinElmer Autosystem XL, USA) equipped with a flame ionization detector (FID) and connected to a HP-Innowax capillary column (30 m × 0.25 mm × 0.25 μm; J & W Scientific). The content of methyl ester obtained was calculated according to European regulated procedure EN14103.

**Table 4:** Physicochemical properties and characteristic of palm oil and Jatropha oil

**Properties (unit)**	**Palm oil**	**Jatropha oil**
Specific gravity (gcm^−3^)	0.860–0.90	0.914
Viscosity at 40°C (cSt)	44.2	54.8
Sulphated Ash (% mass)	0.02	0.0012
Flash point (°C)	182	235
Cloud point (°C)	15	2
Pour point	15	2
Cetane Number	-	46.3
Saponification number (mg g^−1^)	198.9	186.48–193.32
Free Fatty acids% (Kg Kg^−1^ × 100)	<1	9.0–12.0
Fatty acid composition (%)		
Palmitic acid (16:0)	44.2	13.8
Stearic acid (18:0)	4.5	6.8
Oleic acid (18:1)	39.6	41.7
Linoleic acid (18:2)	9.8	35.6

## Conclusions

Heterogeneous base catalysts (NaOH/Al_2_O_3_, MgO, CaO, SrO, CaMgO and CaZnO) were used as catalysts for the production of biodiesel from edible palm and non-edible Jatropha oils. All the base catalysts showed the presence of strong basic strength on the active site except for MgO which contained dominant amount of medium basic sites. The transesterification activity of the heterogeneous base catalysts are correlated with basicity and basic strengths of the catalysts. The optimum conditions for solid base catalysts to achieve >90% of palm biodiesel is 3 wt.% of catalyst, 15:1 of methanol to oil molar ratio within 3 h. Jatropha biodiesel yield required higher amount of catalyst (4 wt.%) and longer reaction time (4 h) under reflux temperature to achieve yield > 80%. It is reasonable to conclude that the type of feedstock oil and chemical characteristic of solid base catalyst strongly affects the yield of biodiesel. Despite of good transesterification activity for solid base catalyst, a good candidate of basic catalyst should be able to tolerate free fatty acids and moisture content in feedstock oil. High catalyst’s reusability without leaching of active component is another desirable characteristic in good candidate catalyst. Calcium-based mixed metal oxides catalysts (CaMgO and CaZnO) render high durability characteristic in transesterification with low active metal leaching for several cycles. The strong interactions between active metals provide a superior synergism of high basicity and stability effect for transesterification reaction.

## Competing interests

The authors declare that they have no competing interests.

## Authors’ contributions

LHV conceived of the study, participated in its design and coordination and drafted the manuscript. JJC provided advice on the testing method, analyzed results and helped to draft the manuscript. NFA and RNM performed partial experiments and analyzed results. YHTY contributed to conception and catalyst design, reaction study and provided research advice in data interpretation. All authors read and approved the manuscript.

## Authors’ information

First Author: Hwei Voon Lee.

Co-Authors: Joon Ching Juan, Nurul Fitriyah binti Abdullah, Rabiah Nizah MF, Yun Hin Taufiq-Yap.

## References

[B1] Abdullah AZ Salamatinia B Mootabadi H Bhatia S Current status and policies on biodiesel industry in Malaysia as the world’s leading producer of palm oil Energ Pol 2009 37 12 5440 5448 10.1016/j.enpol.2009.08.012

[B2] Sharma YC Singh B Upadhyay SN Advancements in development and characterization of biodiesel: a review Fuel 2008 87 12 2355 2373 10.1016/j.fuel.2008.01.014

[B3] Yan S DiMaggio C Mohan S Kim M Salley SO Simon Ng KY Advancements in heterogeneous catalysis for biodiesel synthesis Top Catal 2010 53 721 736 10.1007/s11244-010-9460-5

[B4] Benjumea P Agudelo J Agudelo A Basic properties of palm oil biodiesel-diesel blends Fuel 2008 87 10–11 2069 2075

[B5] Jha SK Fernando S To SDF Flame temperature analysis of biodiesel blends and components Fuel 2008 87 10–11 1982 1988

[B6] Leung DYC Wu X Leung MKH A review on biodiesel production using catalyzed transesterification Appl Energy 2010 87 4 1083 1095 10.1016/j.apenergy.2009.10.006

[B7] Meng X Yang J Xu X Zhang L Nie Q Xian M Biodiesel production from oleaginous microorganisms Renew Energy 2009 34 1 1 5 10.1016/j.renene.2008.04.014

[B8] Bryan RM Biodiesel production, properties and feedstocks Vitro Cell Dev Biol Plant 2009 45 229 266 10.1007/s11627-009-9204-z

[B9] Lim S Lee KT Recent trends, opportunities and challenges of biodiesel in Malaysia: an overview Renew Sust Energ Rev 2010 14 3 938 954 10.1016/j.rser.2009.10.027

[B10] Vasudevan PT Fu B Environmentally sustainable biofuels: advances in biodiesel research Waste Biomass Valorization 2010 1 47 63 10.1007/s12649-009-9002-1

[B11] Basiron Y The Palm Oil Advantage in Biofuel. New Straits Times New Straits Times 24 February 2007. Available from [http://www.mpoc.org.my/The_Palm_Oil_Advantage_In_Biofuel.aspx]

[B12] Wikipedia Table of Biofuel Crop Yields 2009

[B13] Sumathi S Chai SP Mohamed AR Utilization of oil palm as a source of renewable energy in Malaysia Renew Sust Energ Rev 2008 12 9 2404 2421 10.1016/j.rser.2007.06.006

[B14] Sarin R Sharma M Sinharay S Malhotra RK Jatropha-palm biodiesel blends: an optimum mix for Asia Fuel 2007 86 10–11 1365 1371

[B15] Rathore V Madras G Synthesis of biodiesel from edible and non-edible oils in supercritical alcohols and enzymatic synthesis in supercritical carbon dioxide Fuel 2007 86 17–18 2650 2659

[B16] Juan JC Kartika DA Wu TY Hin T-YY Biodiesel production from jatropha oil by catalytic and non-catalytic approaches: an overview Bioresour Technol 2011 102 2 452 460 10.1016/j.biortech.2010.09.093 21094045

[B17] Taufiq-Yap YH Lee HV Pogaku R, Sarbatly RH Higher grade biodiesel production by using solid heterogeneous catalysts Advances in Biofuels 2013 US: Springer 153 176

[B18] Gao L Teng G Xiao G Wei R Biodiesel from palm oil via loading KF/Ca-Al hydrotalcite catalyst Biomass Bioenergy 2010 34 9 1283 1288 10.1016/j.biombioe.2010.03.023

[B19] Zabeti M Daud WMAW Aroua MK Biodiesel production using alumina-supported calcium oxide: an optimization study Fuel Process Technol 2010 91 2 243 248 10.1016/j.fuproc.2009.10.004

[B20] Soetaredjo FE Ayucitra A Ismadji S Maukar AL KOH/bentonite catalysts for transesterification of palm oil to biodiesel Appl Clay Sci 2011 53 341 346 10.1016/j.clay.2010.12.018

[B21] Salamatinia B Mootabadi H Bhatia S Abdullah AZ Optimization of ultrasonic-assisted heterogeneous biodiesel production from palm oil: a response surface methodology approach Fuel Process Technol 2010 91 5 441 448 10.1016/j.fuproc.2009.12.002

[B22] Trakarnpruk W Porntangjitlikit S Palm oil biodiesel synthesized with potassium loaded calcined hydrotalcite and effect of biodiesel blend on elastomer properties Renew Energy 2008 33 7 1558 1563 10.1016/j.renene.2007.08.003

[B23] Hameed BH Lai LF Chin LH Production of biodiesel from palm oil (Elaeis guineensis) using heterogeneous catalyst: an optimized process Fuel Process Technol 2009 90 4 606 610 10.1016/j.fuproc.2008.12.014

[B24] Wan Z Hameed BH Transesterification of palm oil to methyl ester on activated carbon supported calcium oxide catalyst Bioresour Technol 2011 102 3 2659 2664 10.1016/j.biortech.2010.10.119 21109428

[B25] Cho YB Seo G High activity of acid-treated quail eggshell catalysts in the transesterification of palm oil with methanol Bioresour Technol 2010 101 22 8515 8519 10.1016/j.biortech.2010.06.082 20621469

[B26] Baroutian S Aroua MK Raman AAA Sulaiman NMN Potassium hydroxide catalyst supported on palm shell activated carbon for transesterification of palm oil Fuel Process Technol 2010 91 11 1378 1385 10.1016/j.fuproc.2010.05.009

[B27] Viriya-empikul N Krasae P Puttasawat B Yoosuk B Chollacoop N Faungnawakij K Waste shells of mollusk and egg as biodiesel production catalysts Bioresour Technol 2010 101 10 3765 3767 10.1016/j.biortech.2009.12.079 20079632

[B28] Arzamendi G Campo I Arguiñarena E Sánchez M Montes M Gandía LM Synthesis of biodiesel with heterogeneous NaOH/alumina catalysts: comparison with homogeneous NaOH Chem Eng J 2007 134 1–3 123 130

[B29] Kim H-J Kang B-S Kim M-J Park YM Kim D-K Lee J-S Lee K-Y Transesterification of vegetable oil to biodiesel using heterogeneous base catalyst Catal Today 2004 93–95 315 320

[B30] Patil PD Deng S Transesterification of camelina sativa oil using heterogeneous metal oxide catalysts Energy and Fuels 2009 23 9 4619 4624 10.1021/ef900362y

[B31] Fraile JM García N Mayoral JA Pires E Roldán L The basicity of mixed oxides and the influence of alkaline metals: the case of transesterification reactions Appl Catal A Gen 2010 387 1 67 74

[B32] Albuquerque MCG Santamar-Gonzez J Mida-Robles JM Moreno-Tost R Rodruez-Castell E Jimez-Lez A Azevedo DCS Cavalcante CL Jr Maireles-Torres P MgM (Al and Ca) oxides as basic catalysts in transesterification processes Appl Catal A Gen 2008 347 2 162 168 10.1016/j.apcata.2008.06.016

[B33] Di Serio M Ledda M Cozzolino M Minutillo G Tesser R Santacesaria E Transesterification of soybean oil to biodiesel by using heterogeneous basic catalysts Ind Eng Chem Res 2006 45 9 3009 3014

[B34] Lee HV Taufiq-Yap YH Hussein MZ Yunus R Transesterification of jatropha oil with methanol over Mg–Zn mixed metal oxide catalysts Energy 2013 49 12 18

[B35] Hattori H Solid base catalysts: generation, characterization, and catalytic behavior of basic sites J Jpn Petrol Inst 2004 47 2 67 81 10.1627/jpi.47.67

[B36] Kouzu M Yamanaka S-Y Hidaka J-S Tsunomori M Heterogeneous catalysis of calcium oxide used for transesterification of soybean oil with refluxing methanol Appl Catal A Gen 2009 355 1–2 94 99

[B37] Mathiyazhagan M Ganapathi A Factors affecting biodiesel production Res Plant Biol 2011 1 2 1 5

[B38] Babu NS Sree R Prasad PSS Lingaiah N Room-temperature transesterification of edible and nonedible oils using a heterogeneous strong basic Mg/La catalyst Energy Fuels 2008 22 3 1965 1971 10.1021/ef700687w

